# Blood urea nitrogen to creatinine ratio and long-term survival in patients with chronic heart failure

**DOI:** 10.1186/s40001-023-01066-x

**Published:** 2023-09-14

**Authors:** Yajiao Wang, Xia Xu, Shuqing Shi, Xiya Gao, Yumeng Li, Huaqin Wu, Qingqiao Song, Bingxuan Zhang

**Affiliations:** 1 Guang ’anmen Hospital, Chinese Academy of Traditional Chinese Medicine, Beijing, China; 2 Beijing University of Chinese Medicine, Beijing, China

**Keywords:** Chronic heart failure, BUN to SCr ratio, All-cause mortality, Prognosis

## Abstract

**Objectives:**

To explore the correlation between Blood urea nitrogen to creatinine ratio (BUN/Scr ratio) and prognosis of patients with chronic heart failure complicated with renal injury.

**Methods:**

A retrospective analysis of 504 patients hospitalized in Guang 'anmen Hospital, Chinese Academy of Traditional Chinese Medicine from March 2006 to June 2014 was conducted. The baseline data were analyzed, and the cutoff value was obtained by receiver operator characteristic curve (ROC) analysis, according to the cutoff value, all the participants were divided into two groups, BUN/Scr < 19.37 group (280 cases) and BUN/Scr ≥ 19.37 group (224 cases). The main end point was defined as all-cause death. The long-term mortality of the two groups was evaluated, and Kaplan–Meier survival curve was drawn. Univariate analysis was performed on all the variables affecting the patient's prognosis, and the variables with *P* < 0.05 were put into Cox regression model, and subgroup analysis was performed on the variables that might affect the patient’s prognosis.

**Results:**

The baseline data of 504 patients were analyzed and found that the median follow up was 683. Through ROC analysis of 504 subjects, the cutoff value of BUN/Scr was 19.37. The results of Kaplan–Meier survival curve showed that the mortality rate of patients with ratio ≥ 19.37 was higher than that of patients with ratio < 19.37. After multivariate analysis, COX regression model showed that the mortality of patients with BUN/Scr ≥ 19.37 was 1.885 times that of patients with BUN/Scr < 19.37 [HR = 1.885 (1.298–2.737), *P* = 0.001]. Subgroup analysis showed that the relationship between BUN/Scr and the prognosis of CHF was influenced by NYHA and eGRF (*P* < 0.05).

**Conclusions:**

BUN/Scr ratio is related to the poor prognosis of patients with CHF, and is an independent predictor of all-cause death.

## Introduction

Chronic heart failure (CHF) is a clinical syndrome of cardiac output and/or elevated intracardiac pressures at rest or during stress due to structural and/or functional cardiac abnormality [1]. The prevalence of heart failure increases with age: from around 1% for those aged < 55 years to > 10% in those aged 70 years or over [2], which had become a global clinical and public health challenge.

CHF patients often have complications [3], the most important comorbidity was kidney damage [4]. As early as 1836, Robert Bright had decribed the interdependent relationship between heart and kindey [5], with the continuous development of research, the mechanism of kidney disease in patients with heart failure (HF) is gradually concluded to be mostly related to the hemodynamic interactions, neurohormonal activation and so on [6]. In the meantime, blood urea nitrogen (BUN) and serum creatinine (Scr) are the classic indicators of renal function. Nevertheless, BUN is not an accurate index of renal function because excess protein intake, body fluid depletion, heart failure, increased catabolism, and use of diuretics increase BUN levels [7], besides, Scr has been shown to underestimate renal function [8]. In brief, BUN and Scr alone have their limitations. Fortunately, BUN/Scr ratio are recognized indicators of renal insufficiency and there are a few previous reports have shown that the BUN/Scr ratio is associated with prognosis of CHF and is an independent predictor of all-cause mortality [7, 9, 10]

The insufficient renal perfusion, due to the dysfunction of ventricular systolic and/ or diastolic function in patients with CHF, leads to pre-renal acute kidney injury. Activation of the sympathetic nervous system and the renin–angiotensin–aldosterone system (RAAS) decreased urea excretions, and activation of neurohormones increased urea absorption, while creatinine could pass freely through the glomerulus without absorption, increasing BUN/Scr. On the contrary, intrinsic renal disease is the irreversible nephron loss, urea clearance rate, and glomerular filtration rate were decreased simultaneously, resulting in normal BUN/Scr [11]. BUN/Scr can better reflect renal function and evaluate the prognosis of patients with CHF.

In consequence, we conducted the retrospective study to explore the relationship between the BUN/Scr ratio and the prognosis of patients with HF complicated with renal injury.

## Methods

### Study design and eligibility criteria

Consecutive admissions from March 2006 to June 2014 to the cardiology department at the Guang’anmen Hospital affiliated to China Academy of Chinese Medical Sciences with an admitting diagnosis of CHF were reviewed. The diagnosis of CHF was based on the 2005 ACC/AHA guideline update for the diagnosis and management of CHF in the adult [12]. All patients in this study were aged 35 to 95 years old with signs and symptoms related to CHF. Inclusion required an admission N-terminal pro brain natriuretic peptide (NT—pro BNP) level of > 900 pg/ml within 24 h of admission and (or) initial echocardiography assessment left ventricular ejection fraction (LVEF) value of ≤ 50% after hospitalization, and dilated cardiomyopathy (DCM) or ischemic cardiomyopathy (ICM) as the primary cause of CHF, and the New York Heart Association (NYHA) class II to IV category. Patients with the following diseases were excluded: severe cardiac function impairment such as severe valvular disease, acute coronary syndrome, acute pericarditis, cardiogenic shock, malignant arrhythmia, on renal replacement therapy, admitted to interventional cardiology services (to avoid confounding from contrast nephropathy), liver and biliary tract disease, acute renal failure, thyroid dysfunction, active gastrointestinal bleeding, malignant tumors, cognitive impairment, severe mental illness, uncontrolled systemic diseases and the follow-up period less than 30 days.

### Data collection and definitions

Demographic characteristics, medical history, the course of CHF, biochemical, and other laboratory indicators, NYHA category, electrocardiograph and transthoracic echocardiography parameters were collected from electronic medical records at admission. Estimated glomerular filtration rate (eGFR) was calculated by the chronic kidney disease (CKD) Epidemiology Collaboration (CKD-EPI) equation [13]. BUN was calculated by the following formula: BUN (mg/dL) = urea (mmol/L) × 2.8. Information about each event was collected from hospital records or from the testimony of relatives.

### Follow up and endpoints

After discharge, patients were followed up by outpatient electronic medical records system or follow-up telephone calls, all patients were followed up until August 2015. Treatment measures were not interfered during the hospitalization and follow up. The primary endpoint was defined as all-cause mortality during follow-up.

### Statistical analysis

A receiver-operating characteristic (ROC) curve was used to determine the predictive power of BUN/Scr ratio on CHF all-cause mortality, and select the cutoff value to group. According to the cutoff value, patients were divided into two groups. SPSS 25.0 software and MedCalc 19.5.2 Statistical software were used for statistical processing. Measurement data conforming to normal distribution were expressed as mean standard deviation (mean ± sd), and the independent sample *t* test was used for comparison between groups. Measurement data not conforming to normal distribution were expressed as median (upper quartile, lower quartile) [M(Q1, Q3)]. Kruskal–Wallis H test was used for comparison between groups. Enumerative data were expressed by the number of cases (percentage), and the chi-square test was used for comparison. Kaplan–Meier survival curve was used to analyze the survival rate after grouping according to the tangent point values, compare the differences between groups by the log-rank test. Univariate and multivariate Cox proportional risk regression model were used to analyze the correlation between different ratios and the long-term prognosis of CHF. *P* < 0.05 indicates that the difference is statistically significant.

## Results

### Baseline characteristics of study participants

A total of 504 patients were enrolled in this study, the median age of participants was 76 (IQR 70–81) years old with the majority being male (51.5%). The median follow up was 683 (IQR 440.5–730) days. The vast majority of participants had NYHA class 3 or 4 at study enrollment with the most common comorbidities being coronary heart disease (83.3%), (see Table 1). The median eGFR at baseline was 63.9 (IQR 48.6–79.0) ml/min/1.73 m^2^ and the median ratio of BUN/Scr was 18.5 (IQR 15.0–23.5).

**Table 1:** Baseline characteristics of the study

Clinical characteristics	*n* = 504
Age (years)	76 (70–81)
Male Sex, *n* (%)	260 (51.5)
course of disease (years)	6 (2–15)
Follow-up date (days)	683 (440.5–730)
NYHA Class, *n* (%)	
II	76 (15)
III	230 (45.6)
IV	198 (39.2)
LVEF, *n* (%)	
≥ 50%	175 (34.7)
40–49%	211(41.9)
< 40%	118 (23.4)
BUN (mmol/L)	6.9 (5.3–9.1)
Creatinine (μmol/L)	91 (72–113)
BUN/Scr	18.5 (15.0–23.5)
eGFR(ml/min/1.73 m^2^)	63.9 (48.6–79.0)
Comorbidities, *n* (%)	
Coronary heart disease	420 (83.3)
Diabetes	189 (37.5)
Hypertension	373 (74)
Hyperlipidaemia	280 (55.5)

### Predictive ability of BUN/Scr to all-cause mortality in patients with CHF

**Fig. 1:**
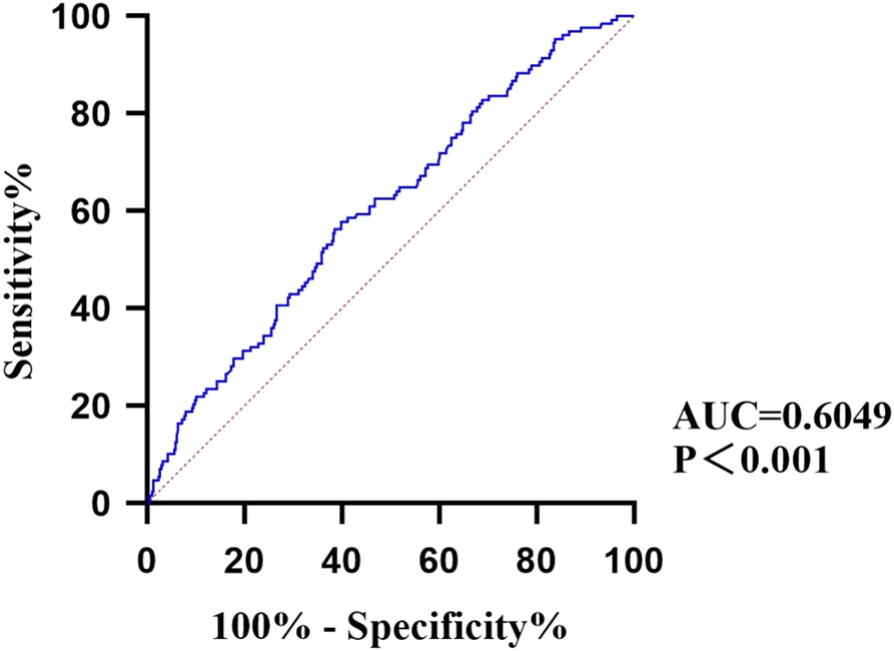
Receiver operating characteristic curves to predict longterm mortality for CHF patients

ROC curve was used to evaluate the predictive ability of BUN/Scr on all-cause mortality in patients with CHF. The results showed that the area under the ROC curve of BUN/Scr was 0.6049, and the cutoff values was 19.37 (Fig. 1).

### Baseline data of the two groups

According to the cutoff value, patients were divided into BUN/Scr < 19.37 group and BUN/Scr ≥ 19.37 group, baseline data of the two groups were analyzed (see Table 2). The differences in eGFR, Scr, BUN, NYHA, NT-proBNP, and coronary heart disease between the 2 groups were statistically significant (*P* < 0.05). There was no statistical significance in age, gender, other complications, and other aspects between the two groups (*P* > 0.05).

**Table 2:** Baseline characteristics and their association with the blood urea nitrogen/Creatinine Ratio

	< 19.37 group (*n* = 280)	≥ 19.37 group (*n* = 224)	*P* value
Age (years)	77 (71, 81)	76 (68, 81)	0.384
Male, *n* (%)	151 (53.9%)	109 (48.7%)	0.448
Course of the disease (years)	6 (2, 14)	6 (2, 16)	0.473
eGFR (ml/min/1.73 m^2^)	60.4 ± 20.2	67.4 ± 23.6	0.007
SCr (mg/dl)	95 (76, 114)	85.0 (68, 111)	< 0.01
BUN (mg/dl)	5.8 (4.6, 7.6)	8.4 (6.7, 11.1)	< 0.01
LVEF (%)	50 (40, 50)	50 (40, 50)	0.961
NT-proBNP (pg/ml)	2183 (1346, 4744.5)	3700 (1846.3, 7701.8)	< 0.01
Coronary heart disease, *n* (%)	253 (90.4%)	167 (74.5%)	< 0.01
Diabetes, *n* (%)	103 (36.8%)	86 (38.4%)	0.711
Hypertension, *n* (%)	207 (73.9%)	166 (74.1%)	0.964
Hyperlipidemia, *n* (%)	160 (57.1%)	120 (53.6%)	0.423
NYHA, *n* (%)	–	–	0.001
II	52 (18.6%)	24 (10.7%)	–
III	134 (47.9%)	96 (42.9%)	–
IV	94 (33.6%)	104 (46.4%)	–

### BUN/Scr on survival time of patients with CHF

**Fig. 2:**
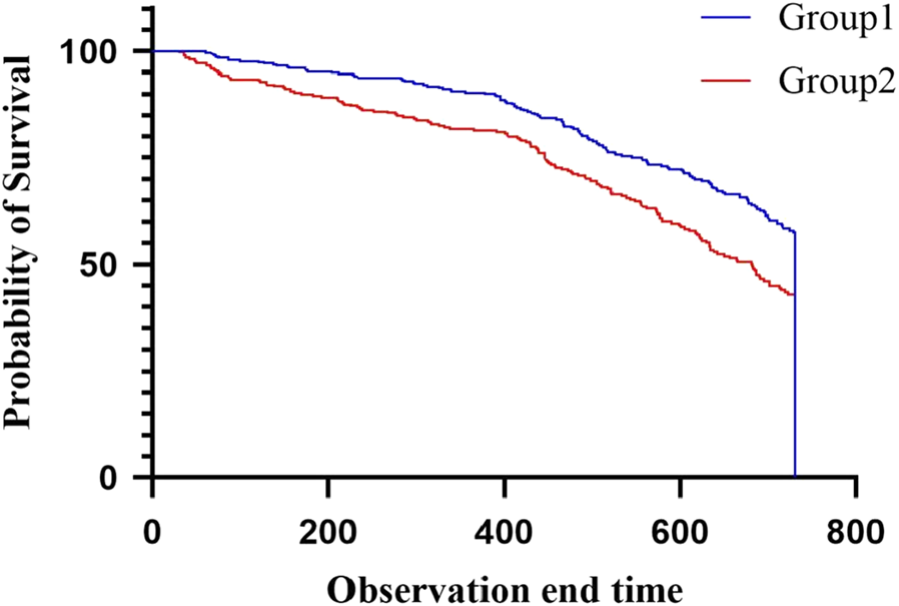
Kaplan–Meier survival curves in patients with different BUN/Scr ratios. Group1: patientswith BUN/Scr < 19.37, Group2: patients with BUN/Scr ≥ 19.37

There were 54 deaths in the group < 19.37 and 75 deaths in the group ≥ 19.37. Then Kaplan–Meier survival curve analysis was conducted. The results show that the survival curve of BUN/Scr ≥ 19.37 was significantly reduced (Fig. 2). The difference between groups was statistically significant (log-rank test: *P* = 0.0002).

### Correlation between BUN/Scr and all-cause death in patients with CHF

All-cause death was used as the dependent variable and BUN/Scr as the independent variable to conduct univariate Cox regression analysis (see Table 3). The results showed that age (HR = 1.025, 95% CI 1.008, 1.043, *P* < 0.005), NYHA (HR = 1.671, 95% CI: 1.323, 2.114, *P* < 0.005) and BUN/Scr (HR = 0.560, 95% CI 0.410, 0.765, *P* < 0.005) were the risk factors of all death in CHF patients. After adjusting with the age, gender, LVEF, NYHA, multivariate Cox regression model showed that BUN/Scr was still an independent risk factor for all-cause death in CHF patients (HR = 0.578, 95%CI 0.416, 0.804, *P* < 0.005).

**Table 3:** Multivariate COX PROPORTIONAL regression analyses of predictive value of the BUN/Scr ratio on all-cause mortality in the CHF population

Variable	Univariate		multivariate	
	HR (95% CI)	*P* value	HR (95% CI)	*P* value
age	1.020 (1.001,1.040)	0.036	1.038 (1.016, 1.060)	0.000
male	1.640 (1.146, 2.346)	0.007	1.801 (1.127, 2.664)	0.003
LVEF	1.109 (0.017,0.713)	0.021	0.260 (0.031, 2.196)	0.216
NYHA	1.575 (1.210,2.051)	0.001	1.644 (1.221, 2.213)	0.001
coronary heart disease	0.937 (0.586,1.496)	0.784	0.785 (0.455, 1.353)	0.383
diabetes	1.220 (0.859,1.733)	0.266	1.276 (0.861, 1.890)	0.224
Hypertension	0.850 (0.578,1.250)	0.409	0.794 (0.524, 1.203)	0.276
Hyperlipidemia	0.859 (0.608,1.215)	0.391	0.730 (0.504, 1.056)	0.094
BUN/Scr ≥ 19.37	2.025 (1.425,2.878)	0.000	1.885 (1.298, 2.737)	0.001

### Subgroup analysis of BUN/Scr ratio in patients with CHF

**Fig. 3:**
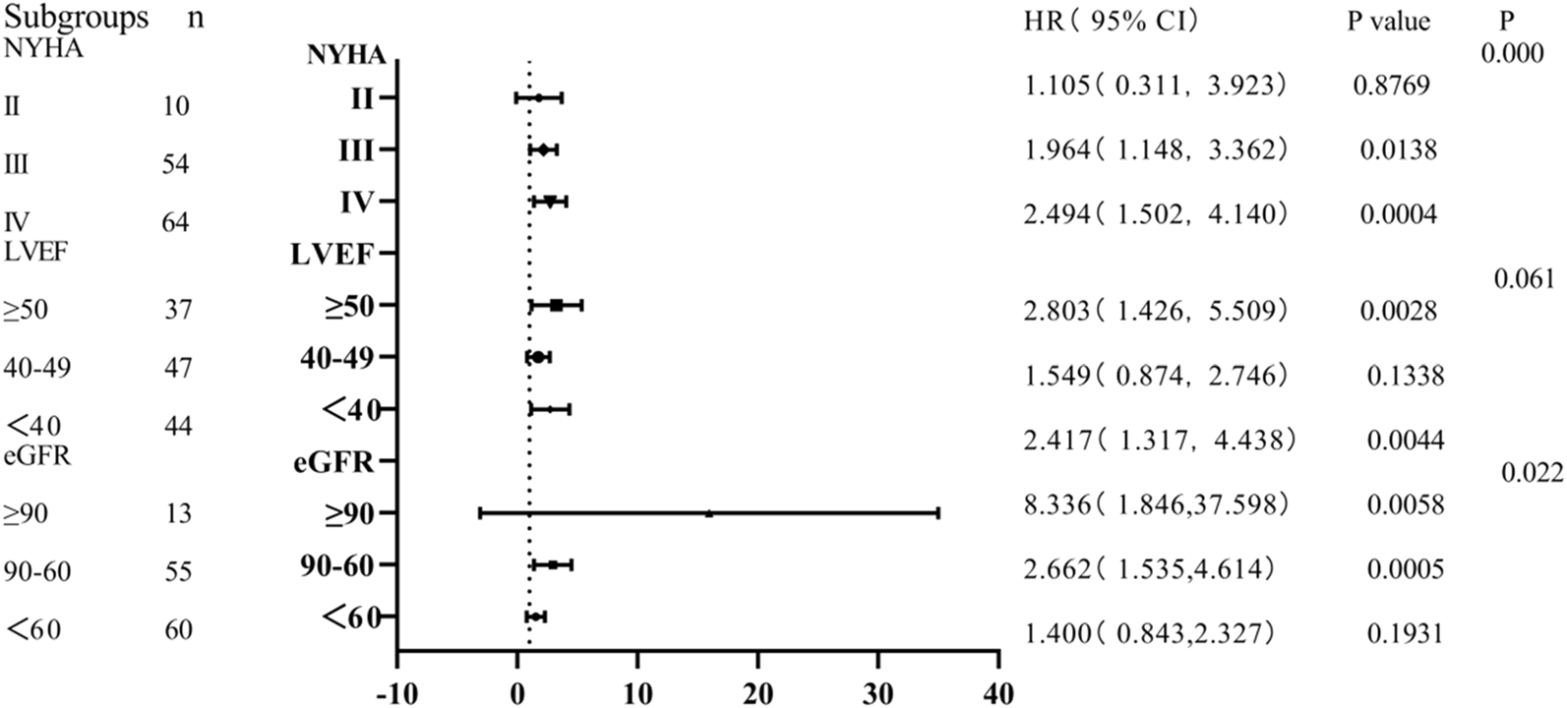
Subgroup analysis forest map. There is significant interaction between NYHA and eGFR

Figure 3 shows that the mortality rate of patients with BUN/Scr ≥ 19.37 is 1.105 times that of patients with ratio < 19.37 in NYHA grade 2, but the difference is not statistically significant [HR = 1.105 (0.311–3.923),* P* = 0.8769]. And that of patients with BUN/Scr ≥ 19.37 is 1.964 times that of patients with ratio < 19.37 in NYHA grade 3. Among patients with NYHA grade 4, the mortality rate of patients with BUN/Scr ≥ 19.37 was 2.494 times that of patients with ratio < 19.37 [HR = 2.494 (1.502–4.140), *P* < 0.05]. The interaction between them was statistically significant (*P* < 0.05). Similarly, the interaction between the ratio and eGFR is statistically significant. The relationship between BUN/Scr and CHF prognosis was not affected by LVEF (*P* > 0.05).

## Discussion

The retrospective analysis of this study concluded that: the results of this study reveals that BUN/Scr ratio is associated with all-cause mortality, and BNU/Scr ≥ 19.37 as the best cutoff points for all-cause mortality, and also associated with higher mortality even after adjustment for other prognostic factors, could be a convenient marker for clinical work.

Scr and BUN are two important indicators for clinical evaluation of renal function. Scr is the product of creatine metabolism with a small molecular weight, most of which passes through glomerular filtration, and almost all of the Scr formed in the body can be excreted by urine. Reduced cardiac output and decreased blood volume due to diuretic use, resulting in renal insufficiency, decreased glomerular filtration rate, increased Scr. BUN is absorbed by renal tubular filtration, in patients with heart failure, decreased cardiac output and insufficient arterial filling lead to the release of sympathetic nervous system (SNS) and RAAS, and increased sodium reabsorption in proximal renal tubules, resulting in increased urea concentration. Activation of SNS is a major cause of cardiac dysfunction and vascular injury and can significantly worsen the prognosis [14, 15]. In addition, insufficient arterial filling leads to the release of baroreceptor mediated arginine vasopressin (AVP) and upregulates the urea transporter in the intramedullary collecting tube. In addition, creatinine is not reabsorbed, which causes a disproportionate increase in BUN and Scr [16].

Considering these mechanisms, elevated BUN/Scr level on admission reveals activation of neurohormones and deterioration of renal function, elevated BUN/Scr ratio is associated with poor prognosis in patients with CHF and is an independent predictor of all-cause mortality. This may be because this ratio represents decreased cardiac output, reduced circulating blood volume, insufficient renal perfusion, unstable hemodynamics, and poor prognosis. There are some similarities between our results and those of others.

According to the median ratio, 557 acute decompensated heart failure patients were divided into high BUN/Scr ratio group and low BUN/Scr ratio group, and the relationship between ratio and long-term mortality was evaluated. During the median follow-up period of 1.9 years, patients with high ratio had higher mortality (*P* = 0.006) [17]. Matsue et al. showed that the median BUN/Scr ratio was 15.0 in 4484 general population without cardiovascular complications, and 21.2 in 2033 patients with acute heart failure. Moreover, the increased ratio was related to more severe heart failure symptoms, and the mortality rate was higher [18]. Kaplan–Meier survival analysis of a prospective cohort study showed that all-cause mortality was higher in patients with a higher BUN/Scr ratio (*P* < 0.0001) [19]. In our study, the tangent value of 19.37 was found by ROC curve at first, and then verified and analyzed by Kaplan−Meier survival curve. The results showed that the survival rate of patients with BUN/Scr ratio ≥ 19.37 was lower than that of patients with BUN/Scr ratio < 19.37.

Multiple studies showed that the BUN/Cr value was still statistically significant after adjusting for multiple factors by COX analysis [20–22]. Even after adjustment for the clinical model including both BUN and Scr, higher than normal range of BUN/Scr ratio group was an independent predictor for all-cause death [HR = 1.86 (1.29–2.66)] [18]. Hao Qian et al. conducted a prospective observational study, in which patients with HF complicated with AMI were included and followed up for 1 year to evaluate the predictors related to mortality. After multivariate COX hazard analysis, BUN/Cr > 15.34 was still < 0.05 [23]. In univariate analysis, the all-cause mortality of BUN/Scr ratio ≥ 25.09 is 90% higher than that of the group with ratio < 25.09 [HR = 1.90 (1.30–2.77)]. Further analysis using multivariate Cox proportional hazard regression, after adjusting the baseline variables (including age, current smokers, LVEF < 40%, atrial fibrillation, MAP, sodium, uric acid, albumin, cystatin C, hemoglobin, RDW, D-dimer, free triiodothyronine, log NT-pro BNP, eGFR, NYHA functional grade, the results showed that BUN/Scr ratio ≥ 25. 09 group [HR = 1.52 (1.03–2.24)] is an independent predictor of long-term death of AHF patients [9]. Similarly, after adjusting the multivariate, this study found that the ratio ≥ 19.37 was significantly related to all-cause mortality in patients with chronic heart failure [HR = 1.885 (1.298, 2.737)], which was consistent with previous research results.

NYHA is a valuable clinical tool, which is related to the potential severity of heart disease and the medium and long-term mortality [24]. In this study, a subgroup analysis of long-term mortality was conducted. The results of BUN/Scr in evaluating the long-term prognosis of patients with chronic heart failure were influenced by NYHA. Furthermore, among patients with IV grade, the prognosis of patients with high BUN/Scr is worse. Among the three subgroups of NYHA, BUN/Scr is statistically significant in predicting the long-term prognosis of patients with CHF (interaction *P* < 0.05).

Many studies have shown that eGFR is a powerful prognostic indicator of CHF [25, 26]. The results of this study show that BUN/Scr has statistical significance in predicting the long-term prognosis of chronic heart failure among the three subgroups of eGFR (Interaction *P* = 0.022).

This study has some limitations. First, it was a retrospective observational study conducted at a single center, with a selection bias due to the data availability, and does not represent all patients with HF. Second, since this study was observational in nature, other confounding factors affecting the results cannot be excluded even after adjusting the analysis. Despite these limitations, our study emphasizes that CHF patients with BUN/Scr ≥ 19.37 at admission have poorer long-term outcomes, and has predictive value for the prognosis of patients with CHF.

## Conclusion

In conclusion, this retrospective analysis indicates that BUN/Cr ratio ≥ 19.37 in HF patients at admission can be used as a simple and effective predictor of all-cause mortality.

## Data Availability

The datasets used and/or analysed during the current study are available from the corresponding author on reasonable request.

## Abbreviations

BUN: Blood urea nitrogen
Scr: Serum creatinine
ROC: Receiver-operator characteristic curve
CHF: Chronic heart failure
HF: Heart failure
RAAS: Renin–angiotensin–aldosterone system
LVEF: Left ventricular ejection fraction
DCM: Dilated cardiomyopathy
ICM: Ischemic cardiomyopathy
NYHA: New York Heart Association
CKD: Chronic kidney disease
SNS: Sympathetic nervous system
AVP: Arginine vasopressin
